# Elucidation and application of the neuroimmune axis between depression and autoimmune diseases: A genome wide and cohort study

**DOI:** 10.1371/journal.pone.0336109

**Published:** 2025-11-10

**Authors:** Fu Sun, Shirong Hui, Bin Gong, Jinhua Gu, Peng Huang

**Affiliations:** 1 The People’s Hospital of Danyang, Affiliated Danyang Hospital of Nantong University, Zhenjiang, China; 2 Department of Epidemiology, Center for Global Health, School of Public Health, Nanjing Medical University, Nanjing, China; 3 The Affiliated Taizhou People’s Hospital of Nanjing Medical University, Taizhou School of Clinical Medicine, Nanjing Medical University, Nanjing, China; Shanghai Jiao Tong University, CHINA

## Abstract

The neuroimmune axis, a critical communication network between the nervous and immune systems, has been implicated in the comorbidity of depression (DeP) and autoimmune diseases (ADs). The shared genetic basis underlying these conditions, and its application are the focus of our investigation, using genome-wide association studies (GWAS) summary statistics and cohort data. We performed a comprehensive analysis, integrating genome-wide and local genetic correlations, casual gene and pleiotropic loci identification, and functional annotation. To improve the power of the identification of shared genes and loci, we proposed the local strategy that the sequential analysis was only in the significantly correlated regions. We defined significant genetic overlap between DeP and ADs, particularly idiopathic pulmonary fibrosis (IPF), rheumatoid arthritis (RA), and ulcerative colitis (UC), with shared loci and genes, such as rs7171171 and *PLCL1*. With the casual relationship, we further defined the higher prediction performance of polygenic risk scores (PRS) for asthma when incorporating DeP. Besides the main analyses, we conducted various sensitivity analyses and side analyses to ensure the robustness of our results. This study not only advances our understanding of the genetic interplay between neuropsychiatric and autoimmune disorders but also provides a framework for future research aimed at targeted therapeutic interventions.

## Introduction

The neuroimmune axis, a vital communication network between the nervous and immune systems, plays a significant role in the pathophysiology of various complex disorders [1–3]. This axis is particularly relevant when studying conditions like depression (DeP) and autoimmune diseases (ADs), which often exhibit comorbidity [4–6]. This comorbidity suggests the presence of shared genetic and biological mechanisms, indicating that neuroimmune interactions might contribute to the development and progression of both DeP and ADs [7,8].

Genome-wide association studies (GWAS) have enhanced our understanding of the genetic underpinnings of DeP and ADs, and provided a substantial foundation for examining their shared genetic basis [9–11]. In parallel, various strategies have been proposed to explore the shared genetic basis, such as transcriptome-wide association studies (TWAS) and Mendelian randomization (MR) [10,12]. Previous studies have not adequately addressed the genetic correlation between DeP and a broad range of ADs or explored the functional implications of shared genetic variants [13–16]. Few studies have systemically addressed the comorbidity between DeP and multiple ADs, leaving an essential aspect of the neuroimmune axis insufficiently explored [11,17,18].

To overcome the limitations, we aim to investigate the shared genetic basis between DeP and 12 ADs and to construct a prediction model utilizing GWAS summary statistics. We include summary statistics from large-scale cohorts for DeP and ADs. To alleviate the multiple comparison issue, we propose a novel local analysis strategy, incorporating local genetic correlations and subsequent pleiotropic analyses, including TWAS, colocalization, and enrichment analysis. Using functional annotation tools, we aim to understand the biological relevance of shared genetic variants and their potential function on disease mechanisms. Finally, we leverage DeP to construct PRS for ADs, with improved prediction performance.

## Methods

### GWAS summary statistics

We collected the summary statistics of DeP from three European cohorts: iPSYCH including 294,322 cases and 74,438 controls [19], FinnGen (version R11) having 53,313 cases and 394,756 controls [20], and 23andMe recruiting 75,607 cases and 231,747 controls [21,22]. Additionally, we included 21 summary statistics for 12 ADs (Table S1 in S1 File). All cohort data and GWAS resources were approved by relevant ethics committees, and written informed consent was obtained from all participants. For each summary statistics, we performed careful quality control (QC), including excluding single nucleotide polymorphisms (SNPs) with minor allele frequency (MAF) < 1%. Given that 11 of the datasets were constructed in GRCh38, we utilized the liftOver tool to convert and harmonize them to GRCh37 [23].

### Meta-analysis

For DeP, adjusting for sample overlap, we first used Multi-Trait Analysis of GWAS (MTAG) to conduct meta-analyses of iPSYCH and FinnGen (*r*_*g*_ = 0.8, *P* < 1.00 × 10^−300^) with the *-equal_h2* option [24]. As the *λ*_*meta*_ between MTAG DeP and 23andMe was 1.13 [25,26], we used the inverse variance weighted (IVW) method in METAL to perform the additional meta-analysis of 23andMe data (Table S2 in S1 File) [27]. For each AD, we similarly used the IVW fixed-effects method in the METAL when there was no sample overlap. When sample overlap was present, we firstly used Metasoft to calculate the heterogeneity index (*I*^*2*^) and determined *P* values based on Cochran’s Q test (*P*_het), then we prioritized the *P* values from meta-analysis using the random-effects model in RE2C when heterogeneity was observed (*I*^*2*^ ≥ 50 or *P*_het < 0.05) [27–29].

For each GWAS meta-analysis result, we used *MungeSumstats* in linkage disequilibrium score regression (LDSC) (version 1.0.1) for rapid standardization and QC [30]. The GWAS obtained from all meta-analyses were evaluated for genetic inflation factor (λ), LDSC intercept, and effective sample size ($\frac{4}{[\frac{1}{n_{case}}+\frac{1}{n_{control}}]}$), as detailed in Fig S1 and Table S2 in S1 File.

### Genome-wide and local genetic correlations

Genome-wide genetic correlations between DeP and 12 ADs were estimated using LDSC and Genetic Covariance Analyzer (GNOVA) [31,32], setting significance at *P* < 4.17 × 10^−3^ and excluding the major histocompatibility complex (MHC) region.

We performed stratified LDSC (s-LDSC) to define the enrichment of 509 cell types, including brain tissue cells (involving neurons cells including neurons, neuroblasts, interneurons, and precursor cells, as well as major glial subtypes including microglia, astrocytes, and oligodendrocytes cells) from Zeisel et al. (N = 39), immune cells from ImmGen (N = 292), and multiple cell types from Franke et al. (N = 27) and Roadmap (N = 151) [33,34]. We excluded non-protein-coding genes, genes with duplicate names, genes located in the MHC region, and genes that were not expressed in any cell type. We then scaled gene expression to a total of 1 million unique molecular identifiers (UMIs) per cell type and calculated the total proportion of each gene expressed in all cell types specific to each cell type. We used top 10% genes in each cell type and estimated the enrichment with adjusting FDR.

For local genetic correlation, among 2,495 regions (including MHC regions) with an average size of 1 Mb. Local Analysis of Variance Annotation (LAVA) was used to define the regions with significant heritability in both trait and correlation at *P* < 0.05/2,495 for both phenotypes [35]. Additionally, pairwise GWAS (GWAS-PW) was used to validate LAVA results, applying a threshold of posterior probability of A3/4 (PP.A3/4) > 0.8 [36]. In subsequent pleiotropic analyses, we focused only on significant genomic regions in the pairs with significant genome-wide genetic correlations. This targeted approach largely reduced multiple comparisons.

### Localized pleiotropic identification and functional enrichment

Shared genetic variants between DeP and ADs were identified through trans-disease meta-analysis (TDMA) and further validated using Pleiotropic Locus Exploration and Interpretation using Optimal Test (PLEIO), taking into account genetic correlations, heritabilities, and environmental covariates. [37,38] We used equally weighted combinations of effect sizes ($\beta_{Trait1,Trait2}=\frac{\beta_{Trait1}+\beta_{Trait2}}{2}$ for shared effects) and variances ($V_{Trait1,Trait2}=\frac{V_{Trait1}+V_{Trait2}}{4}$) [37]. We prioritized these variants with significant SNPs in both TDMA and PLEIO. We used PLINK1.9 to identify independent genome-wide significant pleiotropic loci (*r*^*2*^ less than 0.01 and 500-kilobase [kb] window) [39]. Notably, we considered the MHC region as a locus.

For these potential pleiotropic loci (including all markers in the ± 100 kb range), we performed Bayesian colocalization analysis tests. We used the default settings of the *coloc.abf* function (p1 = p2 = 1.0 × 10^−4^, p12 = 1.0 × 10^−5^; where p1 and p2 represent the prior probabilities of SNPs being associated with trait 1 and trait 2, respectively, and p12 represents the prior probability of SNPs being associated with both traits), and considered a posterior probability of H4 (PP.H4) greater than 0.7 as the criterion for colocalized loci inclusion [40]. This sequential approach ensures that the identified pleiotropic loci are robust and relevant for both DeP and ADs.

The functional impact of shared genetic signaling was investigated using GWAS Analysis of Regulatory or Functional Information Enrichment with LD correction (GARFIELD), focusing on SNPs identified in TDMA [41]. GARFIELD is a powerful enrichment tool that compares 997 different sets of annotated markers from specific cell types and adjusts for LD, MAF, and distance to transcription start sites [41]. Following Patrick et al. [37], we combined the relevant tissues into 26 categories with Bonferroni correction and then investigated the proportion of significant annotations in each category.

### Localized pleiotropic genes identification

To identify hypothetical functional genes with statistical associations to single traits, we used Functional Summary-Based Imputation (FUSION) to define the associated genes and Summary-based MR (SMR) to define the causal genes [42,43]. We used FUSION and SMR, with a Bonferroni-adjusted *P* < 0.05 and Heterogeneity in Dependent Instruments (HEIDI) *P* > 0.01 as inclusion criteria.

For the two methods, we used SNP weights of eQTL from 22 tissues in GTEx v8 (13 brain tissues, adrenal gland, 2 colon tissues, 3 esophagus tissues, lung, pituitary, and thyroid). In addition, for FUSION analysis, we included SNP weights from brain tissues in the CMC, peripheral blood from the NTR, whole blood from the YFS, and whole blood from the METSIM study [44,42]. For SMR analysis, we incorporated SNP weights from BrainMeta brain tissues, blood from the eQTLGen consortium, and 9 brain cell types from 2 different studies [45–50].

Additionally, Polygenic Priority Score (PoPS), a similarity-based gene prioritization method, were employed to pinpoint associations at loci identified in cross-trait meta-analyses [51,52]. PoPS combines GWAS summary statistics with gene expression data, biological pathways, and predicted protein-protein interaction data from more than 50,000 features. We used MAGMA to calculate gene-level association statistics and gene-gene correlations [52], then analyzed the enrichment of 79 gene features. And we determined the PoPS for each gene by fitting a joint model that considered all resulting feature enrichments. We used a POPS Score >1 as the inclusion criterion for the final pleiotropic gene.

### Localized pleiotropic genes validation

To further assess the differential expression of pleiotropic genes in the corresponding diseases, we obtained gene expression profiles for specific diseases, including asthma, DeP, RA, UC, and asthma patients with DeP, from datasets GSE93272, GSE94648, GSE58430, and GSE39653 (Table S3 in S1 File) [53–56]. Specifically, GSE93272 contains RA and control samples, GSE94648 includes UC and controls, GSE58430 comprises asthma with DeP (DeP Asthma), asthma without DeP (No-DeP Asthma), only DeP, and controls, and GSE39653 provides DeP and control samples. The rank sum test was used to assess gene expression differences between diseased and control groups, and the KS test was used to assess expression differences between multiple groups.

### MR

To investigate the bidirectional causality among the 12 trait pairs, we used the IVW method as the primary approach and validated it with MR-Egger, weighted median, and weighted mode [57]. To assess the validity of our instrumental variables (IVs), we conducted heterogeneity and pleiotropy tests. We assessed heterogeneity across instrumental SNPs using Cochran’s Q statistic under both the IVW and MR-Egger models and using the MR-Egger intercept to assess horizontal pleiotropy. We further applied leave-one-out (LOO) analysis to assess whether the causal estimates were driven by any single SNP. In addition, we conducted other sensitivity analyses specific to AD traits: (i) latent heritable confounder Mendelian randomization (lhcMR) to address sample overlap issues; [58] (ii) MR excluding MHC to evaluate robustness; and (iii) multi-variable MR analysis to adjust four potential confounding risk factors: educational attainment, heart rate (including heart rate variability), and body mass index [59].

With InSIDE (Instrument Strength Independent of Direct Effect) assumption, we defined IVs (LD *r*^2^ < 0.001, *P*-value < 1.0 × 10^−8^, and window size = 10 Mb). When the number of IVs was less than three, we relaxed the threshold to *P* < 5.0 × 10^−6^. We then used the phenotypic variance explained by genetic variation (PVE) and the *F*-statistic to assess the strength of genetic association of instrumental SNPs and the problem of weak instrumental bias. For the IVW model, we chose the FDR for multiple testing correction, with a significance threshold of *q* < 0.05.

### Prediction model

Building on the comorbidity and causal relationship, we constructed an AD prediction model using multi-trait Polygenic Score (mtPGS) [60], with single-trait DBSLMM serving as a negative control [61]. We employed PGSFusion to fit both models and evaluated their predictive performance in a cohort of 50,000 individuals from the UK Biobank (UKB) [62]. We evaluated whether the inclusion of Dep information enhances AD prediction in the test dataset by logistic regression models adjusting for age, sex, PGS, and the first 20 genetic principal components, reporting the area under curve (AUC) and its 95% CIs. We conducted multiple comparisons chi-square tests across multiple PGS strata to examine differences in disease prevalence.

Meanwhile, we performed additional analysis to investigate whether the comorbidity could improve the performance of DeP. We also compared the AUC and its 95% CI of mtPGS and DBSLMM.

### Side analysis for meta-AD

To ensure the robustness of the application of the neuroimmune axis for shared genetic basis, we performed the MR and PRS analysis with the meta-analysis of AD (AD-meta). We used the same meta-analysis strategy above to conduct a cross-trait meta-analysis of the eight ADs [29]. We performed MR to elucidate the casual relationship between DeP and AD-meta. To compare the prediction performance, we used AD-meta to construct PRS model with mtPGS and DBSLMM. Specifically, to investigate the scalability of PRS model, we used two kinds of case definitions: (i) defining the AD cases as the individuals suffering from any kind of AD (AS: N = 20; asthma: N = 336; CD: N = 185; CeD: N = 134; IPF: N = 76; PsA: N = 94; RA: N = 268; and UC: N = 409); (ii) defining the asthma cases as the individuals suffering from asthma.

### Statistical analysis

The MHC region (chr6: 28477797–33448354) has complex LD structure and highly correlates to ADs. Different from the previous GWASs, we did not exclude the MHC region and treated it as a single locus when identifying independent SNPs. We constructed a LD panel from 503 European individuals in the 1000 Genome Project phase 3. All statistical analyses were conducted in R version 4.3.0 (R Foundation).

## Results

### Study design

**Fig 1:**
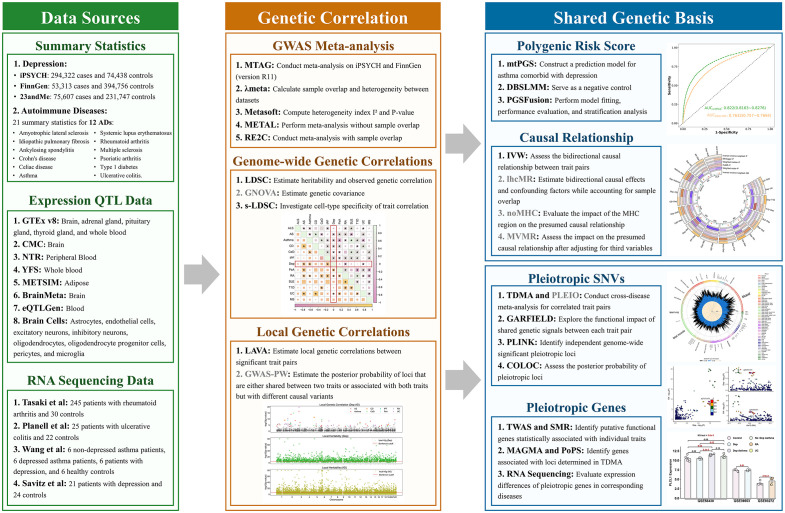
Study design. Summary for the main analyses and sensitivity analyses. The methods with gray font indicate sensitivity analyses.

We collected 21 GWAS summary statistics to elucidate the shared genetic basis between DeP and 12 ADs (amyotrophic lateral sclerosis (ALS), ankylosing spondylitis (AS), asthma, Crohn’s disease (CD), celiac disease (CeD), idiopathic pulmonary fibrosis (IPF), multiple sclerosis (MS), psoriatic arthritis (PsA), rheumatoid arthritis (RA), systemic lupus erythematosus (SLE), type 1 diabetes (T1D), and ulcerative colitis (UC) [10,20–22,63–70]. For the traits with multiple summary statistics, we performed meta-analysis considering the sample overlapping and heterogeneity. Besides summary statistics, we also involved expression quantitative trait locus (eQTL) from genotype tissue expression (GTEx) for pleiotropic gene identification and RNA sequencing (RNA-seq) for pleiotropic gene validation. Genetic correlations were explored at both the genome-wide and local levels. Based on the correlated regions, we identified pleiotropic SNPs and genes associated with DeP and ADs through cross-trait meta-analysis, and functional annotation. Finally, using the causal relationships, we constructed PRS to predict comorbid conditions, emphasizing the shared genetic etiology and its implications for understanding the neuroimmune axis. Specifically, to enhance the robustness of our study, we additionally performed sensitivity analysis and side analysis for each procedure. Details are shown in Fig 1.

### DeP meta-analysis

Based on DeP meta-analysis, encompassing an effective sample size of 1,292,933 individuals and 8,277,496 SNPs, we estimate heritability and intercept, yielding values of 0.0471 (*P* < 1 × 10^−300^) and 1.17, respectively. The genomic inflation factor (*λ*_*meta*_) was 1.76, indicating that the observed inflation was due to polygenicity, consistent with previous findings (Table 1) [19].

**Table 1:** Summary for the DeP and 12 ADs with Meta-analysis.

Trait	Sample Size	*λ*	Intercept	*h* ^2^	P.*h*^2^
Depression	1 292 933	1.76	1.1711 (0.0116)	0.0471 (0.0016)	1.00 × 10^-300^
ALS	152 268	1.09	1.0275 (0.0072)	0.0346 (0.0039)	1.00 × 10^-300^
AS	325 798	1.05	1.0458 (0.0109)	0.0097 (0.0029)	8.23 × 10^−4^
Asthma	319 091	1.21	1.073 (0.0136)	0.0842 (0.006)	1.00 × 10^-300^
CD	31 366	1.05	1.0239 (0.0085)	0.1139 (0.0211)	6.73 × 10^−8^
CeD	27 031	1.05	1.006 (0.0069)	0.09 (0.0214)	2.60 × 10^−5^
IPF	14 172	1.02	1.0016 (0.0065)	0.0778 (0.0313)	1.29 × 10^−2^
MS	180 491	1.05	1.0314 (0.0087)	0.0161 (0.0035)	4.22 × 10^−6^
PsA	266 381	1.06	1.0304 (0.009)	0.0157 (0.0026)	1.56 × 10^−9^
RA	102 572	1.10	1.0459 (0.0126)	0.1587 (0.0139)	1.00 × 10^-300^
SLE	32 375	1.03	1.0101 (0.0088)	0.0529 (0.021)	1.18 × 10^−2^
T1D	90 531	1.25	1.2102 (0.0143)	0.2181 (0.0234)	1.00 × 10^-300^
UC	50 079	1.11	1.0762 (0.0112)	0.2896 (0.0254)	1.00 × 10^-300^

This table summarizes the key metrics from GWAS meta-analyses for DeP and 12 ADs, including effective sample sizes, genomic inflation factors (*λ*), intercept (standard error), heritability (*h*^2^) (standard error), and *P*-value (P.*h*^2^). The heritability (*h*^2^) reflects the proportion of variance in each trait attributable to genetic factors. The intercept values are indicative of population structure or cryptic relatedness.

### Genome-wide genetic correlations

**Fig 2:**
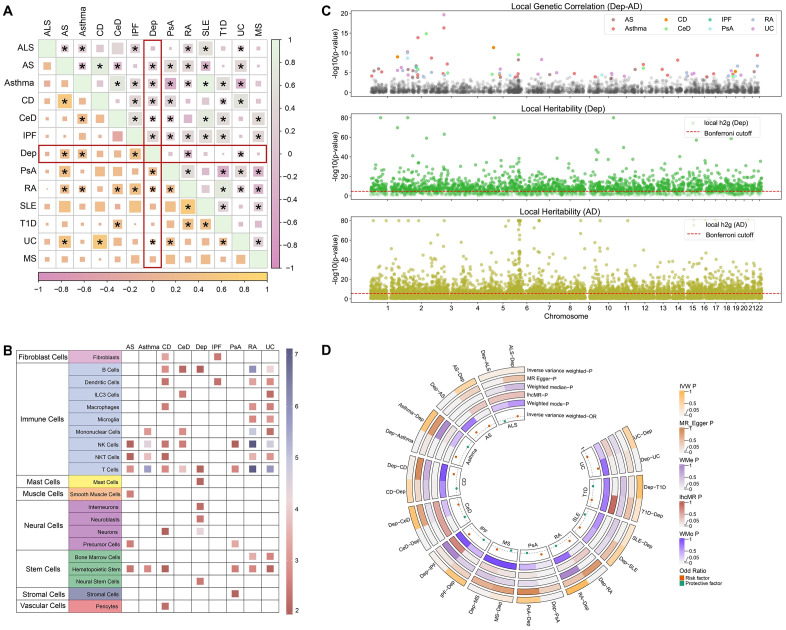
Genome-wide and local genetic correlation between DeP and ADs. **A.** The heatmap illustrates the genetic correlations between various traits, with the bottom left displaying results from LDSC and the top right from GNOVA. Each cell represents the genetic correlation, with significant correlations marked by asterisks. **B.** The matrix shows the results of the stratified s-LDSC analysis. It includes a total of 509 cell types from four datasets, with the most specific 10% of genes in each cell type selected for downstream analysis. Each cell represents the Z-value, with colors ranging from red (low Z-value) to purple (high Z-value). **C.** The top plot shows local genetic correlations between DeP and various autoimmune diseases. The middle plot displays local heritability for DeP, while the bottom plot illustrates local heritability for autoimmune diseases, with the Bonferroni cutoff indicated by a dashed red line. Significant regions are highlighted and color-coded by traits. **D.** The circular plot illustrates the bidirectional MR results between DeP and autoimmune diseases. Orange dots represent risk factors, while green dots represent protective factors. Various MR methods are used, including IVW, MR-Egger, Weighted Median, Weighted Mode, and lhcMR, with significance thresholds indicated.

Among the 12 paired traits comparisons, eight pairs exhibited significant genome-wide genetic correlations in at least one model. These pairs were used for subsequent analyses (Fig 2A and Table S4 in S1 File). Notably, five shared trait pairs were between DeP and AS, asthma, IPF, RA, and UC. The strongest correlation was observed for IPF (LDSC: *r*_*g*_ = 0.35, *P* = 3.0 × 10^−4^; GNOVA: *r*_*g*_ = 0.37, *P* = 1.1 × 10^−12^), in line with the previous epidemiological evidence [71]. The s-LDSC analyses of DeP defined the significantly associated cell types were predominantly neurons (*P* = 1.5 × 10^−5^). However, all glial cell types did not show nominal enrichment signals (Fig 2B and Table S5 in S1 File), likely due to the relatively smaller sample sizes of glial-specific eQTL studies compared to immune cell datasets. Eight ADs primarily defined various immune cells (Fig 2B and Table S5 in S1 File). Interestingly, both diseases showed significant associations with B cells and T cells without FDR correction, suggesting a potential shared genetic basis.

### Local genetic correlations

We utilized LAVA to investigate local genetic correlations among eight pairs. A total of 65 regions, including three MHC regions, demonstrated localized genetic correlations between ADs and DeP (Fig 2C). GWAS-PW confirmed 45 regions (Table S6 in S1 File). Among them, two regions stood out due to their significant correlations with multiple trait pairs: region 226 (chr2: 22429641–23538527) and region 291 (chr2: 85992795–86867032). For example, region 291 was significant in three trait pairs: DeP-AS (*P* = 1.9 × 10^−7^), DeP-IPF (*P* = 4.0 × 10^−7^), and DeP-RA (*P* = 4.8 × 10^−6^). Interestingly, different from other pairs, DeP-IPF in region 291 displayed a negative genetic correlation. Using Ensembl, we identified 17 genes in region 291, many of which are associated with mitochondrial function (Table S7 in S1 File) [72]. Among these genes, six were associated with sad facial emotion recognition, and several were implicated in immune system functions, lending additional credibility to our findings [73].

### Localized pleiotropies and enrichment analysis

We identified 1,540 genome-wide significant SNPs from 65 regions across eight trait pairs using TDMA (*P* < 5.0 × 10^−8^). There was a high degree of concordance between PLEIO and TDMA results, with an average significant SNP overlap of 72% in each trait pair. Notably, for the DeP-RA and DeP-UC, the overlap rate exceeded 95% (Table S8 in S1 File). Using LD aggregation, we identified 17 pleiotropic SNPs distributed across four trait pairs (Table S8 in S1 File). These had fewer *P* values in TDMA than those in a single trait and showed the same effect direction.

**Fig 3:**
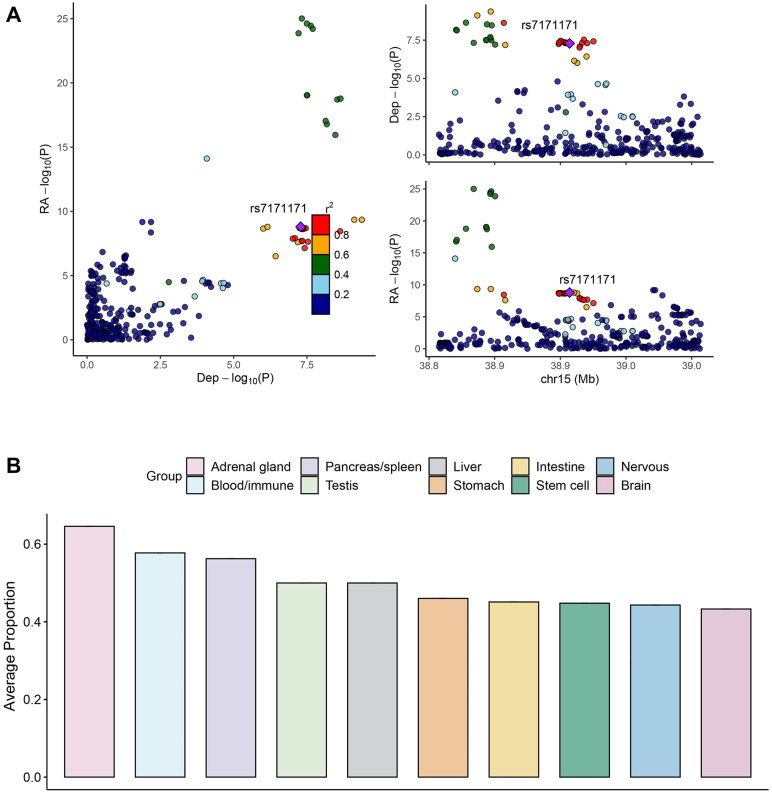
Result of colocalization and functional enrichment analysis. **A.** This panel illustrates the colocalization results for DeP and RA. The left plot shows a scatter plot of -log10(p-values) for depression (x-axis) and RA (y-axis), with each point representing a SNP. The color of each point indicates the linkage disequilibrium (LD) with the top SNP (rs7171171). The top right plot displays the regional association for depression on chromosome 15, and the bottom right plot shows the regional association for RA in the same region, with the top SNP (rs7171171) highlighted. **B.** This bar chart represents the GARFIELD analysis results, showing the average proportion of significant annotations across different tissue groups. The groups include adrenal gland, blood/immune, brain, intestine, liver, nervous, pancreas/spleen, stem cell, stomach, and testis. The proportions indicate the enrichment of functional annotations related to the shared genetic signals between DeP and ADs.

Using colocalization analysis of the 17 pleiotropic SNPs (including all markers within ±100 kb), we identified nine potential pleiotropic SNPs (53%) with PP.H4 > 0.7 (Fig S1 and Table S9 in S1 File). Notably, PP.H4 of rs7171171, an intergenic variant near *RASGRP1*, was 1.0 in the DeP-RA trait pair (Fig 3A). Although rs7171171 was not significant in DeP (*P* = .013), another SNP within the *RASGRP1* was highly significant (*P*_rs56059718_ = 1.0 × 10^−9^). [74,19] This locus is also associated with various ADs, such as UC and T1D [75–77].

We applied GARFIELD to investigate the functional impact of eight traits on shared genetic signaling, focusing on SNPs more significant in each pair’s TDMA than in any individual disease. The results revealed higher proportions of significant annotations in the adrenal gland (65%), thymus (62%), blood/immune (58%), and brain (43%) categories. Enrichment was observed in all categories, suggesting the neuroimmune axis, involving whole-body systems, plays a key role in the common genetic signal (Fig 3B). Excluding the DeP-CeD (19%) pair, the percentage of Blood/immune category increased the to 63%, with DeP-RA contributing the most (96%) (Table S10 in S1 File). The lower percentage in the DeP-CeD pair may result from CeD’s unique genetic architecture and distinct immune mechanisms. Additionally, B cells (*P* = 3.7 × 10^−48^) and monocytes (*P* = 1.4 × 10^−41^) were involved in multiple trait pairs within the Blood/immune category (Fig S3 in S1 File), with mitochondrial DNA (mtDNA) from CD14 + monocytes closely associated with autoimmune diseases [78]. This is consistent with LAVA and s-LDSC results, emphasizing the involvement of ADs and DeP in the immune pathway [79,80].

### Localized pleiotropic genes

Using FUSION, we identified 236 significant genes across 65 regions, with 12 genes shared in three trait pairs. These genes were located across 15 different tissues, including eight brain tissues (Table S11 in S1 File) and associated with both multiple psychiatric and autoimmune disorders (Fig S4 in S1 File) [73]. Notably, in thyroid tissues, we identified a significant regulatory role for the gene *PLCL1* in the comorbidities of both DeP (*P* = 4.29 × 10^−6^) and asthma/RA (*P*_*asthma*_* *= 1.07 × 10^−4^ and *P*_*RA*_ = 3.90 × 10^−3^).

Through SMR validation, we found that *PLCL1* remained a risk factor for the three ADs (Table S12 in S1 File). However, we only found the gene associated with DeP in thyroid tissue (SMR *P* = 1.47 × 10^−5^), whereas in asthma (SMR *P* = 1.38 × 10^−3^) and RA (SMR *P* = 6.52 × 10^−4^), it played a role in dopaminergic neuron function. Although *PLCL1* also had a risk effect on DeP in dopaminergic neurons (*P* = 1.45 × 10^−2^), the HEIDI test suggested a potential heterogeneity (*P* = 1.18 × 10^−3^). In addition, we found that PoPS of *PLCL1* was greater than 1 in the DeP-asthma (Score = 2.62) and DeP-RA (Score = 2.16) trait pairs (Table S13 in S1 File). This result re-emphasizes that *PLCL1* plays a key role in DeP and ADs.

Additionally, we found that the *TRIM8* prominently regulated DeP (Thyroid: FUSION *P* = 4.69 × 10^−5^; Brain: SMR *P* = 1.56 × 10^−5^) and UC (Thyroid: FUSION *P* = 6.88 × 10^−6^; Brain, SMR *P* = 7.28 × 10^−6^) among FUSION, SMR and MAGMA. Although its PoPS Score did not reach the threshold (Score = 0.40), the MAGMA results highlighted a significant association in the trait pair (*P* = 5.03 × 10^−3^).

### Validation of *PLCL1* Using RNA-seq

**Fig 4:**
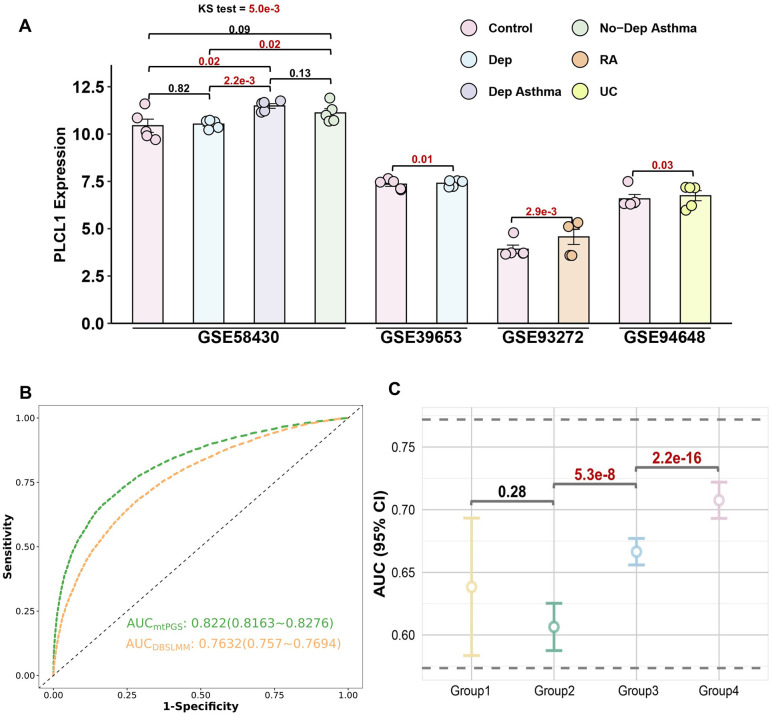
Results for RNA-seq validation of *PLCL1*, causal relationship, and PRS for asthma. **A.** Barplot of *PLCL1* expression levels across different conditions. *PLCL1* expression levels were analyzed in various datasets: GSE58430, GSE39653, GSE93272, and GSE94648. In the first dataset, there were 6 non-depressed asthma (No-DeP Asthma) patients, 6 depressed asthma (DeP Asthma) patients, 6 patients with depression (DeP), and 6 healthy controls. The second dataset included 21 patients with DeP and 24 healthy controls. The third dataset comprised 245 patients with rheumatoid arthritis (RA) and 30 healthy controls. In the fourth dataset, there were 25 patients with ulcerative colitis (UC) and 22 healthy controls. Expression was compared between controls, individuals with DeP, RA, UC, No-DeP Asthma, and DeP Asthma. The Kolmogorov-Smirnov (KS) test was used to determine the statistical significance of differences between groups. Significant differences (*P*-values) are indicated: *P* < 0.05 is denoted in red, demonstrating higher *PLCL1* expression in asthma, RA, UC, and depression groups compared to controls. **B.** Receiver operating characteristic (ROC) curves of polygenic risk scores for Asthma with mtPGS and DBSLMM. The green dashed line represents the ROC for the predictive performance of mtPGS, while the orange dashed line indicates the ROC for DBSLMM. It includes the AUC values and their 95% CIs for both methods. **C.** Ponit plot of the prediction performance of different genetic risk groups. It shows the AUC along with their 95% CIs for four PGS groups divided by 0.1, 0.4, 0.4, and 0.1. We performed roc tests for differences in AUCs between adjacent groups with *P* values.

We further evaluated the expression of *PLCL1* in asthma, RA, or DeP patients and controls. The Kolmogorov-Smirnov test indicated that *PLCL1* expression was higher in the individuals of asthma with DeP, asthma without DeP, and only DeP than that in the control (*P* = 5.0 × 10^−3^, Fig 4A). Although there was no significant difference between the depressive asthma group and the non-depressive asthma group (*P* = 0.13), and no difference from the control group when asthma patients did not have DeP (*P* = 0.09), the difference is significant when asthma was combined with DeP (*P* = 0.02). Additionally, in other datasets, we also detected the differentially expressed *PLCL1* (DeP: *P* = 0.01, RA: *P* = 2.9 × 10^−3^ and UC: *P* = 0.03).

### Single variable and multivariable MR

From the IVW results, we found that DeP was associated with a higher risk of AS, asthma, PsA, RA, and UC, whereas CeD was associated with a lower risk of DeP (Fig 2D and Table S14 in S1 File). We observed heterogeneity in certain trait pairs (Table S15 in S1 File), but the MR-Egger intercept test indicated no evidence of horizontal pleiotropy (*P* > 0.05) (Table S16 in S1 File).

lhcMR validation revealed that, excluding RA, DeP maintained a significant causal relationship with the other four ADs, consistent with the effect direction from the IVW method (Table S17 in S1 File). After excluding the MHC region, the results remained consistent with the lhcMR findings (Table S18 in S1 File). In multi-variable MR, we found that three trait pairs (DeP-AS, DeP-asthma, and DeP-PsA) remained significantly associated after accounting for the confounding factors. Specially, educational attainment weakened the association between DeP and PsA, while all third variables reduced the association between DeP and UC (Table S19 in S1 File).

Through meta-analysis and QC, we retained 2,391,322 SNPs that appeared across all eight ADs (*λ* = 1.23, *h*^²^ = 0.023) (Fig S5 in S1 File). We estimated the genetic correlation between DeP and AD-meta (*r*_*g*_ = 0.84, *P* < 1.00 × 10^−300^). Similarly, in the MR analysis of AD-meta, we observed that DeP increases the risk of ADs, with results confirmed by all sensitivity analyses (OR = 1.71, *P* = 2.05 × 10^-303^). However, in the reverse analysis, we again found no evidence that ADs either increase or decrease the risk of DeP (OR = 2.61, *P* = 0.17).

### Prediction model with PRS

Considering the prevalence, we only constructed a prediction model for asthma (prevalence in UKB = 0.67%), rather than PsA and UC. Firstly, the prediction performance of mtPGS (AUC = 0.82, 95% confidential interval [CI]: 0.82–0.83) is significantly higher than that of DBSLMM (AUC = 0.76, 95% CI: 0.82–0.83) (Fig 4B). Meanwhile, the asthma cannot improve the prediction performance of DeP, with mtPGS (AUC: 0.66, 95% CI: 0.65–0.67) and DBSLMM (AUC: 0.67, 95% CI: 0.66–0.68). Next, using the asthma PRS constructed by mtPGS, we stratified into low (10%), medium (80%), and high (90%) genetic risk groups, respectively. We found the prevalence was 0.16%, 0.44%, and 3.06% with a significant difference (*P* = 6.64 × 10^−105^). Further pairwise comparisons between different PRS groups using Bonferroni correction showed significant differences in asthma prevalence between all pairs (Fig 4C and Table S20 in S1 File). In the reverse PRS analysis, we found that asthma did not significantly enhance the risk prediction for DeP, with the DBSLMM-lmm model (AUC = 0.67, 95% CI: 0.66–0.68) outperforming the mtPGS model (AUC = 0.66, 95% CI: 0.65–0.66) (Fig S6A in S1 File). This result aligns with our MR findings, further supporting the unidirectional causal relationship between DeP and asthma, where genetic susceptibility to DeP increases asthma risk.

In the side analysis, when we used 8 ADs for validation, we observed no significant difference in predictive performance between the mtPGS model (AUC = 0.60, 95% CI: 0.59–0.62) and the single-trait DBSLMM model (AUC = 0.59, 95% CI: 0.58–0.61) (Fig S6B in S1 File). However, when using asthma as the validation trait, we noted a more pronounced improvement in predictive performance with the mtPGS model (AUC = 0.67, 95% CI: 0.67–0.68) compared to the DBSLMM model (AUC = 0.64, 95% CI: 0.63–0.65) (Fig S6C in S1 File).

## Discussion

This study investigated the genome-wide and localized genetic correlations between DeP and ADs, uncovering shared genetic etiology and potential pleiotropy. Our findings provide insights into the biological mechanisms underlying the comorbidity of DeP and ADs, emphasizing the genetic factors in the neuroimmune axis.

Our genetic correlation analysis of large-scale GWAS data for DeP revealed significant genetic correlations with eight out of 12 ADs, particularly IPF, corroborating epidemiological evidence and suggesting a genetic link between DeP and IPF [71,81]. Although the DeP meta-analysis showed an elevated genomic inflation factor (λ = 1.76), the LDSC intercept (1.17) and highly significant SNP-heritability indicate that the majority of this elevation arises from the expected combination of very large sample size and extensive polygenicity. Our study-level QC, overlap adjustment, and random-effects procedures further limit residual confounding, supporting the robustness of downstream analyses. To evaluate the shared genetic architecture between traits while minimizing potential confounding from differences in statistical power, we estimated genome-wide genetic correlations using two complementary approaches (LDSC and GNOVA). Both methods incorporate LD structure and provide diagnostic statistics to assess estimate stability. Importantly, results from GNOVA were fully consistent with those from LDSC, reinforcing the robustness of our findings. In addition, we excluded the MHC region and applied a conservative significance threshold (*P* < 4.17 × 10^−3^) to minimize spurious correlations.

Through local genetic correlation analysis, we identified 65 regions with significant correlations between ADs and DeP. Notably, regions chr2:22429641–23538527 and chr2:85992795–86867032 consistently showed genetic correlations across three trait pairs. This region is closely linked to mitochondrial function, which is crucial in the neuroimmune axis, affecting both neurons and immune cells. In the nervous system, mitochondria support synaptic transmission, plasticity, and neuronal health by regulating calcium balance and producing ATP [82]. In the immune system, mitochondria are involved in cytokine production and immune cell activation [83]. Mitochondrial dysfunction can lead to oxidative stress and inflammation, contributing to psychiatric and autoimmune diseases [84]. The release of mitochondrial DNA (mtDNA) can trigger immune responses, linking neuronal damage to immune activation [85].

Through TDMA, we identified 1,540 genome-wide significant SNPs across eight trait pairs, highlighting 17 pleiotropic loci. Notably, rs7171171, located near *RASGRP1*, underscores a potential role in the genetic interplay between these conditions. *RASGRP1* is crucial for T cell signaling, impacting the MAPK/ERK pathway, and its dysregulation can lead to immune imbalances in autoimmune diseases [86–88]. Additionally, *RASGRP1* plays a crucial role in dopamine signaling and immune response. It may contribute to psychiatric disorders by affecting key brain regions, such as the dorsolateral prefrontal cortex and hippocampus, as well as influencing the inflammatory state in peripheral blood [89]. This immune imbalance is similarly implicated in the development of DeP, where chronic inflammation and excessive inflammatory responses release pro-inflammatory cytokines (e.g., IL-6, TNF-*α*) that affect the central nervous system [90–92]. These findings underscore the interconnected roles of the neuroimmune axis in both DeP and ADs.

We identified 12 pleiotropic genes, including *PLCL1*, *SPRED1*, and *CNNM2*, associated with the comorbidity of DeP and autoimmune diseases. Notably, *PLCL1* plays a significant regulatory role in DeP, asthma, and RA. *PLCL1*, a member of the PLC family, plays a crucial role in cellular signaling by catalyzing the hydrolysis of phospholipid molecules to generate diacylglycerol and inositol trisphosphate [93]. Variants in this gene have been shown to be associated with various psychiatric and ADs [94,95]. Specifically, dysregulation of *PLCL1* affects intracellular signaling networks and stress response pathways, which are crucial for cell growth, survival, and metabolism [96–98]. *PLCL1* not only directly regulates immune cells and inflammatory factors but also impacts the development and progression of autoimmune diseases by interacting with other immune-related pathways. For example, *PLCL1* interacts with the NLRP3 inflammasome, modulating calcium signaling and phospholipid metabolism within immune cells, thereby affecting the intensity and duration of immune responses [95]. This dysregulation can lead to depressive symptoms in neurons and abnormal immune responses in immune cells, highlighting *PLCL1’*s key role in the neuroimmune axis and the interconnected nature of DeP and ADs.

MR analysis provided evidence for the bidirectional causal relationship between DeP and ADs. We observed strong evidence that genetic susceptibility to DeP causally increases the risk of three AD phenotypes, consistent with previous findings [12,99]. Additionally, we discovered an intriguing phenomenon suggesting that genetic susceptibility to CeD might reduce the risk of DeP, although this finding did not hold up in subsequent sensitivity analyses. Previous studies on the relationship between CeD and DeP have shown conflicting results: some suggest a positive correlation [100], while others indicate no difference in DeP risk between CeD patients and the general population [101]. We believe this controversy may be related to adherence to a strict gluten-free diet (GFD). Management through GFD can potentially reduce systemic inflammation, thereby decreasing neuroinflammation associated with DeP [102]. Furthermore, GFD helps restore beneficial gut microbiota balance, enhancing the health of the neuroimmune axis and positively impacting mental health.

PRS have become crucial tools for understanding complex genetic phenotypes and advancing precision medicine. Consistent with previous reports, we found a significant association between DeP PRS and asthma diagnosis [103]. By incorporating DeP PRS, the mtPGS method provided a statistically significant improvement in asthma prediction. This finding supports the notion that DeP genetic information enhances known risk factors for asthma [103]. Our PRS model showed substantial improvement, indicating its potential for effective patient risk stratification and its applicability in future large-scale GWAS.

This study has several limitations. First, the GWAS data utilized primarily involved individuals of EUR, limiting the generalizability of our findings to other ethnic groups. Future research should incorporate more diverse populations to validate and expand upon these results. Second, our analysis focused on common genetic variants and did not consider the potential impact of rare or structural variants, which could also play significant roles in the comorbidity between DeP and ADs. Third, although glial subtypes were included in our analysis, none showed nominal enrichment, likely due to the limited size and resolution of available glial reference datasets. Therefore, while our findings highlight neuronal contributions, they do not exclude potential roles of glial cells, which warrant investigation in future studies with larger and more refined datasets. Fourth, our validation relied mainly on blood-derived transcriptomic data, which only partially overlap with the full range of tissue contexts used in FUSION and SMR, and may not fully capture disease-specific alterations in primary tissues. Thus, the results provide supportive but not tissue-specific evidence for the prioritized pleiotropic genes. Finally, while we employed multiple sensitivity analyses to address pleiotropy and heterogeneity, the inherent limitations of MR and other statistical methods may still influence the robustness of our causal inferences.

Based on local analysis strategy, this study offers innovative insights into the shared genetic etiology of DeP and ADs, emphasizing the neuroimmune axis’s pivotal role. Our findings not only enhance our knowledge of disease etiology but also open new avenues for targeted therapies for individuals with comorbid DeP and ADs.

## Supporting information

S1 File Fig. S1. Quantile-Quantile (Q-Q) plots for Genome-Wide Association Study (GWAS) of various traits. **Fig. S2.** Regional Association Plots. **Fig. S3.** Significant peaks from GARFIELD enrichment analysis. **Fig. S4.** The enrichment of 12 pleiotropic genes using FUMA. **Fig. S5.** Q-Q plots for GWAS meta-analysis for eight ADs. **Fig. S6.** PRS results for AD GWAS meta-analysis. **Table S1**. Details of GWAS summary data sources. **Table S2**. Genetic correlations between iPSYCH and FinnGen. **Table S3**. Detailed information for RNA expression profiling datasets. **Table S4**. Summary for the genetic correlations between DeP and 12 ADs. **Table S5**. Association of DeP and ADs with nominal significance on specific cell types analyzed. **Table S6.** Summary for local genetic correlations in the eight trait pairs. **Table S7.** Gene information of 17 genes on the chr2: 85992795–86867032 region. **Table S8.** Overlap rate of significant SNPs identified by TDMA and PLEIO. **Table S9.** Summary for TDMA and COLOC results for the pleiotropic SNPs. **Table S10.** Functional effects of pleiotropic SNPs from GARFIELD in eight trait pairs. **Table S11.** Summary for the FUSION result of significant genes on 65 regions. **Table S12.** Summary for the SMR result. **Table S13.** Summary for the MAGMA and POPS results for the shared genes. **Table S14.** Summary for the bidirectional MR analysis of 12 trait pairs. **Table S15.** Summary for heterogeneity of bidirectional MR analysis. **Table S16.** Summary for horizontal pleiotropy of bidirectional MR analysis. **Table S17.** Summary for the lhcMR result of bidirectional MR analysis. **Table S18.** Summary for the MR result without the MHC region. **Table S19.** Summary for the MVMR result. **Table S20.** Summary for the pairwise comparisons between PRS groups.
(ZIP)
