# Supplementary material for: Elucidation and application of the neuroimmune axis between depression and autoimmune diseases: A genome wide and cohort study
Source: PLoS One. 2025 Nov 10;20(11):e0336109. doi: 10.1371/journal.pone.0336109 (PMC12599955; doi:10.1371/journal.pone.0336109)
Supplement: S1 File — Fig. S1. Quantile-Quantile (Q-Q) plots for Genome-Wide Association Study (GWAS) of various traits. Fig. S2. Regional Association Plots. Fig. S3. Significant peaks from GARFIELD enrichment analysis. Fig. S4. The enrichment of 12 pleiotropic genes using FUMA. Fig. S5. Q-Q plots for GWAS meta-analysis for eight ADs. Fig. S6. PRS results for AD GWAS meta-analysis. Table S1. Details of GWAS summary data sources. Table S2. Genetic correlations between iPSYCH and FinnGen. Table S3. Detailed information for RNA expression profiling datasets. Table S4. Summary for the genetic correlations between DeP and 12 ADs. Table S5. Association of DeP and ADs with nominal significance on specific cell types analyzed. Table S6. Summary for local genetic correlations in the eight trait pairs. Table S7. Gene information of 17 genes on the chr2: 85992795–86867032 region. Table S8. Overlap rate of significant SNPs identified by TDMA and PLEIO. Table S9. Summary for TDMA and COLOC results for the pleiotropic SNPs. Table S10. Functional effects of pleiotropic SNPs from GARFIELD in eight trait pairs. Table S11. Summary for the FUSION result of significant genes on 65 regions. Table S12. Summary for the SMR result. Table S13. Summary for the MAGMA and POPS results for the shared genes. Table S14. Summary for the bidirectional MR analysis of 12 trait pairs. Table S15. Summary for heterogeneity of bidirectional MR analysis. Table S16. Summary for horizontal pleiotropy of bidirectional MR analysis. Table S17. Summary for the lhcMR result of bidirectional MR analysis. Table S18. Summary for the MR result without the MHC region. Table S19. Summary for the MVMR result. Table S20. Summary for the pairwise comparisons between PRS groups. (ZIP) [file pone.0336109.s001.zip › Supplementary files/Table S10.docx]

**Table S10.** Functional effects of pleiotropic SNPs from GARFIELD in eight trait pairs.

| **Pair** | **Group** | **total_annotations** | **significant_annotations** | **proportion_significant** |
| --- | --- | --- | --- | --- |
| Depression-AS | Adrenal gland | 12 | 8 | 66.67% |
| Depression-AS | Blood/immune | 334 | 141 | 42.22% |
| Depression-AS | Brain | 82 | 5 | 6.10% |
| Depression-AS | Breast | 30 | 11 | 36.67% |
| Depression-AS | Cervix | 42 | 16 | 38.10% |
| Depression-AS | Connective | 82 | 0 | 0.00% |
| Depression-AS | Embryo/uterus | 32 | 9 | 28.13% |
| Depression-AS | Epithelium | 66 | 6 | 9.09% |
| Depression-AS | Eye | 8 | 3 | 37.50% |
| Depression-AS | Heart | 62 | 5 | 8.06% |
| Depression-AS | Intestine | 120 | 33 | 27.50% |
| Depression-AS | Kidney | 72 | 10 | 13.89% |
| Depression-AS | Liver | 62 | 21 | 33.87% |
| Depression-AS | Lung | 180 | 0 | 0.00% |
| Depression-AS | Muscle | 212 | 0 | 0.00% |
| Depression-AS | Nervous | 22 | 0 | 0.00% |
| Depression-AS | Pancreas/spleen | 20 | 9 | 45.00% |
| Depression-AS | Prostate | 16 | 5 | 31.25% |
| Depression-AS | Skin | 88 | 17 | 19.32% |
| Depression-AS | Stem cell | 120 | 37 | 30.83% |
| Depression-AS | Stomach | 44 | 5 | 11.36% |
| Depression-AS | Testis | 8 | 3 | 37.50% |
| Depression-AS | Thymus | 40 | 18 | 45.00% |
| Depression-AS | Urothelium | 8 | 2 | 25.00% |
| Depression-AS | Vascular endothelial | 102 | 20 | 19.61% |
| Depression-Asthma | Adrenal gland | 12 | 12 | 100.00% |
| Depression-Asthma | Blood/immune | 334 | 274 | 82.04% |
| Depression-Asthma | Brain | 82 | 75 | 91.46% |
| Depression-Asthma | Breast | 30 | 24 | 80.00% |
| Depression-Asthma | Cervix | 42 | 31 | 73.81% |
| Depression-Asthma | Connective | 82 | 68 | 82.93% |
| Depression-Asthma | Embryo/uterus | 32 | 20 | 62.50% |
| Depression-Asthma | Epithelium | 66 | 57 | 86.36% |
| Depression-Asthma | Eye | 8 | 8 | 100.00% |
| Depression-Asthma | Heart | 62 | 60 | 96.77% |
| Depression-Asthma | Intestine | 120 | 96 | 80.00% |
| Depression-Asthma | Kidney | 72 | 52 | 72.22% |
| Depression-Asthma | Liver | 62 | 43 | 69.35% |
| Depression-Asthma | Lung | 180 | 149 | 82.78% |
| Depression-Asthma | Muscle | 212 | 163 | 76.89% |
| Depression-Asthma | Nervous | 22 | 22 | 100.00% |
| Depression-Asthma | Pancreas/spleen | 20 | 16 | 80.00% |
| Depression-Asthma | Prostate | 16 | 15 | 93.75% |
| Depression-Asthma | Skin | 88 | 61 | 69.32% |
| Depression-Asthma | Stem cell | 120 | 103 | 85.83% |
| Depression-Asthma | Stomach | 44 | 40 | 90.91% |
| Depression-Asthma | Testis | 8 | 8 | 100.00% |
| Depression-Asthma | Thymus | 40 | 40 | 100.00% |
| Depression-Asthma | Urothelium | 8 | 4 | 50.00% |
| Depression-Asthma | Vascular endothelial | 102 | 77 | 75.49% |
| Depression-CD | Adrenal gland | 12 | 2 | 16.67% |
| Depression-CD | Blood/immune | 334 | 148 | 44.31% |
| Depression-CD | Brain | 82 | 4 | 4.88% |
| Depression-CD | Breast | 30 | 8 | 26.67% |
| Depression-CD | Cervix | 42 | 17 | 40.48% |
| Depression-CD | Connective | 82 | 11 | 13.41% |
| Depression-CD | Embryo/uterus | 32 | 10 | 31.25% |
| Depression-CD | Epithelium | 66 | 7 | 10.61% |
| Depression-CD | Eye | 8 | 1 | 12.50% |
| Depression-CD | Heart | 62 | 6 | 9.68% |
| Depression-CD | Intestine | 120 | 27 | 22.50% |
| Depression-CD | Kidney | 72 | 12 | 16.67% |
| Depression-CD | Liver | 62 | 19 | 30.65% |
| Depression-CD | Lung | 180 | 24 | 13.33% |
| Depression-CD | Muscle | 212 | 33 | 15.57% |
| Depression-CD | Nervous | 22 | 3 | 13.64% |
| Depression-CD | Pancreas/spleen | 20 | 10 | 50.00% |
| Depression-CD | Prostate | 16 | 7 | 43.75% |
| Depression-CD | Skin | 88 | 9 | 10.23% |
| Depression-CD | Stem cell | 120 | 13 | 10.83% |
| Depression-CD | Stomach | 44 | 19 | 43.18% |
| Depression-CD | Testis | 8 | 2 | 25.00% |
| Depression-CD | Thymus | 40 | 23 | 57.50% |
| Depression-CD | Urothelium | 8 | 4 | 50.00% |
| Depression-CD | Vascular endothelial | 102 | 21 | 20.59% |
| Depression-CeD | Adrenal gland | 12 | 7 | 58.33% |
| Depression-CeD | Blood/immune | 334 | 63 | 18.86% |
| Depression-CeD | Brain | 82 | 42 | 51.22% |
| Depression-CeD | Breast | 30 | 3 | 10.00% |
| Depression-CeD | Cervix | 42 | 9 | 21.43% |
| Depression-CeD | Connective | 82 | 1 | 1.22% |
| Depression-CeD | Embryo/uterus | 32 | 1 | 3.13% |
| Depression-CeD | Epithelium | 66 | 2 | 3.03% |
| Depression-CeD | Eye | 8 | 3 | 37.50% |
| Depression-CeD | Heart | 62 | 5 | 8.06% |
| Depression-CeD | Intestine | 120 | 4 | 3.33% |
| Depression-CeD | Kidney | 72 | 6 | 8.33% |
| Depression-CeD | Liver | 62 | 17 | 27.42% |
| Depression-CeD | Lung | 180 | 12 | 6.67% |
| Depression-CeD | Muscle | 212 | 0 | 0.00% |
| Depression-CeD | Nervous | 22 | 8 | 36.36% |
| Depression-CeD | Pancreas/spleen | 20 | 2 | 10.00% |
| Depression-CeD | Prostate | 16 | 0 | 0.00% |
| Depression-CeD | Skin | 88 | 3 | 3.41% |
| Depression-CeD | Stem cell | 120 | 30 | 25.00% |
| Depression-CeD | Stomach | 44 | 2 | 4.55% |
| Depression-CeD | Testis | 8 | 2 | 25.00% |
| Depression-CeD | Thymus | 40 | 1 | 2.50% |
| Depression-CeD | Urothelium | 8 | 1 | 12.50% |
| Depression-CeD | Vascular endothelial | 102 | 12 | 11.76% |
| Depression-IPF | Adrenal gland | 12 | 12 | 100.00% |
| Depression-IPF | Blood/immune | 334 | 169 | 50.60% |
| Depression-IPF | Brain | 82 | 68 | 82.93% |
| Depression-IPF | Breast | 30 | 14 | 46.67% |
| Depression-IPF | Cervix | 42 | 19 | 45.24% |
| Depression-IPF | Connective | 82 | 53 | 64.63% |
| Depression-IPF | Embryo/uterus | 32 | 10 | 31.25% |
| Depression-IPF | Epithelium | 66 | 33 | 50.00% |
| Depression-IPF | Eye | 8 | 7 | 87.50% |
| Depression-IPF | Heart | 62 | 37 | 59.68% |
| Depression-IPF | Intestine | 120 | 45 | 37.50% |
| Depression-IPF | Kidney | 72 | 37 | 51.39% |
| Depression-IPF | Liver | 62 | 35 | 56.45% |
| Depression-IPF | Lung | 180 | 106 | 58.89% |
| Depression-IPF | Muscle | 212 | 62 | 29.25% |
| Depression-IPF | Nervous | 22 | 21 | 95.45% |
| Depression-IPF | Pancreas/spleen | 20 | 12 | 60.00% |
| Depression-IPF | Prostate | 16 | 7 | 43.75% |
| Depression-IPF | Skin | 88 | 43 | 48.86% |
| Depression-IPF | Stem cell | 120 | 80 | 66.67% |
| Depression-IPF | Stomach | 44 | 18 | 40.91% |
| Depression-IPF | Testis | 8 | 8 | 100.00% |
| Depression-IPF | Thymus | 40 | 26 | 65.00% |
| Depression-IPF | Urothelium | 8 | 5 | 62.50% |
| Depression-IPF | Vascular endothelial | 102 | 38 | 37.25% |
| Depression-PsA | Adrenal gland | 12 | 2 | 16.67% |
| Depression-PsA | Blood/immune | 334 | 132 | 39.52% |
| Depression-PsA | Brain | 82 | 2 | 2.44% |
| Depression-PsA | Breast | 30 | 3 | 10.00% |
| Depression-PsA | Cervix | 42 | 13 | 30.95% |
| Depression-PsA | Connective | 82 | 1 | 1.22% |
| Depression-PsA | Embryo/uterus | 32 | 0 | 0.00% |
| Depression-PsA | Epithelium | 66 | 1 | 1.52% |
| Depression-PsA | Eye | 8 | 0 | 0.00% |
| Depression-PsA | Heart | 62 | 0 | 0.00% |
| Depression-PsA | Intestine | 120 | 3 | 2.50% |
| Depression-PsA | Kidney | 72 | 0 | 0.00% |
| Depression-PsA | Liver | 62 | 14 | 22.58% |
| Depression-PsA | Lung | 180 | 2 | 1.11% |
| Depression-PsA | Muscle | 212 | 0 | 0.00% |
| Depression-PsA | Nervous | 22 | 0 | 0.00% |
| Depression-PsA | Pancreas/spleen | 20 | 4 | 20.00% |
| Depression-PsA | Prostate | 16 | 1 | 6.25% |
| Depression-PsA | Skin | 88 | 3 | 3.41% |
| Depression-PsA | Stem cell | 120 | 12 | 10.00% |
| Depression-PsA | Stomach | 44 | 0 | 0.00% |
| Depression-PsA | Testis | 8 | 0 | 0.00% |
| Depression-PsA | Thymus | 40 | 10 | 25.00% |
| Depression-PsA | Urothelium | 8 | 0 | 0.00% |
| Depression-PsA | Vascular endothelial | 102 | 5 | 4.90% |
| Depression-RA | Adrenal gland | 12 | 12 | 100.00% |
| Depression-RA | Blood/immune | 334 | 321 | 96.11% |
| Depression-RA | Brain | 82 | 66 | 80.49% |
| Depression-RA | Breast | 30 | 30 | 100.00% |
| Depression-RA | Cervix | 42 | 33 | 78.57% |
| Depression-RA | Connective | 82 | 82 | 100.00% |
| Depression-RA | Embryo/uterus | 32 | 32 | 100.00% |
| Depression-RA | Epithelium | 66 | 66 | 100.00% |
| Depression-RA | Eye | 8 | 7 | 87.50% |
| Depression-RA | Heart | 62 | 61 | 98.39% |
| Depression-RA | Intestine | 120 | 120 | 100.00% |
| Depression-RA | Kidney | 72 | 72 | 100.00% |
| Depression-RA | Liver | 62 | 56 | 90.32% |
| Depression-RA | Lung | 180 | 175 | 97.22% |
| Depression-RA | Muscle | 212 | 208 | 98.11% |
| Depression-RA | Nervous | 22 | 21 | 95.45% |
| Depression-RA | Pancreas/spleen | 20 | 19 | 95.00% |
| Depression-RA | Prostate | 16 | 15 | 93.75% |
| Depression-RA | Skin | 88 | 88 | 100.00% |
| Depression-RA | Stem cell | 120 | 107 | 89.17% |
| Depression-RA | Stomach | 44 | 44 | 100.00% |
| Depression-RA | Testis | 8 | 7 | 87.50% |
| Depression-RA | Thymus | 40 | 40 | 100.00% |
| Depression-RA | Urothelium | 8 | 8 | 100.00% |
| Depression-RA | Vascular endothelial | 102 | 93 | 91.18% |
| Depression-UC | Adrenal gland | 12 | 7 | 58.33% |
| Depression-UC | Blood/immune | 334 | 295 | 88.32% |
| Depression-UC | Brain | 82 | 22 | 26.83% |
| Depression-UC | Breast | 30 | 27 | 90.00% |
| Depression-UC | Cervix | 42 | 32 | 76.19% |
| Depression-UC | Connective | 82 | 36 | 43.90% |
| Depression-UC | Embryo/uterus | 32 | 27 | 84.38% |
| Depression-UC | Epithelium | 66 | 37 | 56.06% |
| Depression-UC | Eye | 8 | 5 | 62.50% |
| Depression-UC | Heart | 62 | 32 | 51.61% |
| Depression-UC | Intestine | 120 | 105 | 87.50% |
| Depression-UC | Kidney | 72 | 36 | 50.00% |
| Depression-UC | Liver | 62 | 43 | 69.35% |
| Depression-UC | Lung | 180 | 90 | 50.00% |
| Depression-UC | Muscle | 212 | 128 | 60.38% |
| Depression-UC | Nervous | 22 | 3 | 13.64% |
| Depression-UC | Pancreas/spleen | 20 | 18 | 90.00% |
| Depression-UC | Prostate | 16 | 16 | 100.00% |
| Depression-UC | Skin | 88 | 49 | 55.68% |
| Depression-UC | Stem cell | 120 | 48 | 40.00% |
| Depression-UC | Stomach | 44 | 34 | 77.27% |
| Depression-UC | Testis | 8 | 2 | 25.00% |
| Depression-UC | Thymus | 40 | 39 | 97.50% |
| Depression-UC | Urothelium | 8 | 7 | 87.50% |
| Depression-UC | Vascular endothelial | 102 | 56 | 54.90% |
